# Life-Threatening Anemia and Thrombocytopenia in a Toddler with Influenza B: Case Report and Literature Review

**DOI:** 10.3390/children12050632

**Published:** 2025-05-14

**Authors:** Irina Profir, Cristina-Mihaela Popescu, Iuliana Moraru

**Affiliations:** 1 Clinical Medical Department, Faculty of Medicine and Pharmacy, “Dunărea de Jos” University of Galați, 800216 Galați, Romania; irina.profir@ugal.ro (I.P.); iuliana.moraru@ugal.ro (I.M.); 2 Clinical Emergency Children’s Hospital “Sf. Ioan”, 800487 Galați, Romania; 3 Dental-Medicine Department, Faculty of Medicine and Pharmacy, “Dunărea de Jos” University of Galați, 800201 Galați, Romania

**Keywords:** influenza B virus, anemia, thrombocytopenia, respiratory tract infections, pediatrics

## Abstract

**Background**: Seasonal influenza viruses are primarily known for causing respiratory illness, but rare hematologic complications can occur, especially in young children. While influenza A is more commonly linked to severe manifestations, influenza B can similarly precipitate life-threatening cytopenias, particularly in toddlers. **Case Presentation:** We report the case of a previously healthy 1-year-and-8-months-old girl who presented with a high fever, cough, and marked pallor during peak influenza season. Laboratory tests revealed significant microcytic, hypochromic anemia and severe thrombocytopenia. Rapid antigen testing was positive for influenza B. An extensive workup for other causes of bicytopenia, including leukemia, hemolysis, aplastic anemia, and other viral infections, yielded negative results. The child was managed with urgent red blood cell and platelet transfusions, oseltamivir antiviral therapy, broad-spectrum antibiotics, corticosteroids, and supportive care. Bone marrow aspiration was deferred in light of the rapid hematologic recovery. Her hemoglobin greatly improved, and her platelet count reached normal values at discharge. **Conclusions**: Our case underscores the need to consider influenza in the differential diagnosis of unexplained cytopenias during flu season. This case illustrates that influenza B can mimic hematologic malignancies. Rapid diagnosis and supportive treatment are essential to avoid fatal outcomes. Influenza vaccination plays a significant role in preventing severe complications, such as those we encountered.

## 1. Introduction

Influenza remains a significant cause of morbidity in the pediatric population, particularly affecting younger children. Common complications include pneumonia, acute otitis media, and neurologic involvement. Additionally, while these well-documented, rare hematological manifestations are not extensively reported, they are increasingly recognized as part of the broader clinical spectrum [1,2].

Influenza B infection, though historically understudied compared to influenza A, can trigger life-threatening cytopenias in toddlers—a poorly addressed phenomenon in current guidelines [3]. Infections caused by type A influenza viruses are typically more prevalent than those caused by type B [4]. Recent epidemiologic data indicate that influenza A results in more pediatric cases than influenza B [5,6]. Despite its lower incidence, influenza B can still be clinically significant [7,8]. Children (especially infants and toddlers) have higher risks for influenza A- and B-related hospitalization than influenza-associated deaths [3,9,10]. Although influenza B is often considered less severe than influenza A, severe systemic manifestations have been documented even with type B infections [3]. The clinical severity of influenza can vary by strain and season; for instance, one report noted an increased severity of influenza A/H3N2 in children during the 2022 season [11].

While less common than respiratory or neurological manifestations, hematologic complications of influenza represent a significant issue for hospitalized patients [12,13]. Reports of significant anemia are infrequent [13]. Notably, even previously healthy children have occasionally developed substantial cytopenias during influenza infection. However, children with underlying chronic hematologic or immunologic conditions are more susceptible to these complications [14,15]. Influenza B can trigger proinflammatory cytokines (e.g., interferon-γ, tumor necrosis factor-α), which may transiently inhibit hematopoiesis. In the absence of hemolysis [low reticulocyte count, normal lactate dehydrogenase (LDH)/bilirubin, haptoglobin, and uric acid levels, and no hemoglobinuria] and rapid hematologic recovery, cytokine-mediated marrow suppression can be the primary mechanism. This is consistent with prior reports of virus-associated bone marrow suppression [14,15].

Influenza-associated thrombocytopenia may partially be attributed to increased platelet destruction or consumption; platelets can notably internalize influenza virions, leading to platelet activation and clearance [16]. Immune-mediated thrombocytopenia resembling acute immune thrombocytopenic purpura (ITP) was also described during influenza [17]. In rare instances, influenza triggers complement activation and hemolysis, as observed in influenza-associated hemolytic uremic syndrome (HUS) [17]. These observations emphasize that influenza A and B can induce hematologic complications through various immunopathologic mechanisms, affecting even previously healthy children [14,15]. We present a unique case of a toddler with an influenza B infection who developed life-threatening anemia and thrombocytopenia. Current guidelines provide limited direction on the evaluation and management of such cases. By highlighting the potential for severe bone marrow suppression and discussing diagnostic and therapeutic considerations, our case underscores the need for greater clinical awareness of influenza B-associated cytopenias. Our case fills a gap in the pediatric literature on influenza B complications in young children. We correlate our findings with recent literature to discuss possible pathophysiologic mechanisms and to emphasize the importance of recognizing influenza as a potential cause of severe cytopenias in children.

## 2. Case Report

A previously healthy 1-year-and-8-months-old female was brought to the hospital with a 3-day history of high fever (up to 39.4 °C), a dry cough, and an episode of post-tussive vomiting. The parents noted the child’s increasing pallor and lethargy. There was no reported bleeding, rash, toxin exposure, or family history of hematologic disorders.

On examination, the toddler appeared pale and ill but remained alert. Daily clinical evolution is presented in Table 1.

Vital signs revealed a respiratory rate of 40/min and a heart rate of 134/min, with blood pressure measuring 90/48 mmHg. She was febrile (38.7 °C) and exhibited oxygen saturation levels of 95–98% on room air. Her weight (8.1 kg) and length (78 cm) fell below the fifth percentile for age, consistent with chronic undernutrition (ponderal hypotrophy). She was hemodynamically stable (no shock). Notably, there was significant mucosal pallor. No petechiae or purpura were observed on the skin. Lung auscultation revealed mild rhonchi (consistent with viral pneumonitis), but no crackles or wheezing were detected. The liver and spleen were not palpable, and no lymphadenopathy was found. The neurological examination was unremarkable.

The daily evolution of the lab results is depicted in Table 2. 

The initial results indicated significant bicytopenia. Hemoglobin was 3.1 g/dL (markedly low for age, with microcytic, hypochromic indices; standard lower limit ~11 g/dL). MCV was 55 fL (low). PLT was 15 × 10^3^ μL (reference ~150–450 × 10^3^ μL). The WBC count was 5.66 × 10^3^ μL, within the normal range (no leukopenia); the differential showed mild relative lymphopenia. The ARC at admission was 0.68%, with an absolute count of 0.0146 × 10^6^/µL, both inappropriately low for the severity of anemia. Although the percentage is within the standard pediatric reference range (0.5–1.5%), the low absolute value (reference: 0.03–0.12 × 10^6^ μL) suggests an insufficient bone marrow response, making ongoing hemolysis or recovery unlikely at that time. Peripheral smear revealed severe hypochromia (+++), microcytosis, and anisopoikilocytosis, but no schistocytes or blasts, consistent with iron deficiency and viral suppression. Serum ferritin measured 14.6 ng/mL (low, indicating iron deficiency). Inflammatory markers were unremarkable (C-reactive protein < 1 mg/dL, procalcitonin 0.1 ng/mL). The patient’s low MCV and reduced ferritin level were consistent with iron deficiency anemia. Ferritin is a marker of iron stores and an acute-phase reactant, meaning its levels can rise in response to inflammation or infection. Therefore, while low ferritin remains a highly specific indicator of iron deficiency anemia (IDA), especially in the absence of inflammation, normal or even elevated ferritin levels do not reliably exclude IDA when an acute or chronic inflammatory process is present. This diagnostic limitation is particularly relevant in pediatric patients recovering from viral infections, where subclinical inflammation may mask underlying iron deficiency [18,19,20]. A therapeutic trial of oral iron supplementation was recommended upon discharge.

Hemolysis markers were within normal limits, as depicted in Table 3.

The direct antiglobulin (Coombs) test was negative. Coagulation studies were normal (prothrombin time 11.7 s, INR 0.98; activated partial thromboplastin time 19.4 s; fibrinogen 378 mg/dL; D-dimer 0.47 mg/L), indicating no evidence of disseminated intravascular coagulation or thrombotic microangiopathy. Immunoglobulin levels (IgA, IgG, IgM) were age-appropriate, suggesting no underlying immunodeficiency.

Given the combination of severe anemia and thrombocytopenia, a rapid influenza antigen test (nasopharyngeal swab) was performed and was positive for influenza B. Additional virologic tests for hepatitis A, B, and C, parvovirus B19, Epstein–Barr virus, cytomegalovirus, and HIV were all negative. Blood cultures showed no bacterial growth. Bone marrow aspiration was considered due to the unexplained bicytopenia, but initial management priorities were resuscitative and supportive given the child’s critical anemia.

**Figure 1:**
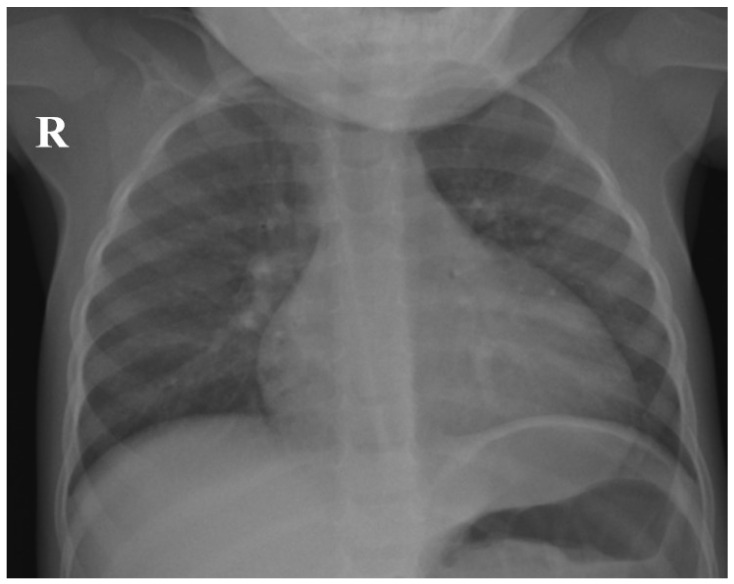
Chest X-ray of the patient demonstrating bilateral peribronchial interstitial shadowing, consistent with viral pneumonitis (R—right side of the patient).

The chest X-ray (Figure 1) taken during hospital admission demonstrated bilateral peribronchial interstitial shadowing, consistent with a viral pneumonitis. The abdominal ultrasound revealed no hepatosplenomegaly or lymphadenopathy.

The child was admitted to the pediatric intensive care unit and treated with a multidisciplinary supportive approach. She received an urgent transfusion of packed red blood cells (10 mL/kg) for the symptomatic severe anemia. A platelet transfusion dose (one pooled unit) was administered prophylactically for the platelet count < 20 × 10^3^ μL to reduce bleeding risk. Although ITP was included in the differential diagnosis, a platelet transfusion was administered due to the extremely low platelet count (15,000/μL), as a precaution to reduce the risk of spontaneous bleeding while awaiting diagnostic clarification. This decision was made despite the absence of active hemorrhage, recognizing that transfusions are typically ineffective in confirmed cases of ITP.

Oseltamivir (3 mg/kg per dose, orally twice daily) was started promptly for confirmed influenza B. Empiric intravenous ceftriaxone was given to cover possible bacterial co-infection while awaiting culture results. Intravenous (IV) hydrocortisone was initiated (given concern for potential hyperinflammation or immune-mediated cytopenias such as influenza-associated hemophagocytic syndrome or ITP) and later transitioned to a short course of IV methylprednisolone. Other supportive care measures included IV maintenance fluids and electrolytes, supplemental vitamins (including folate and a multivitamin), and antipyretics. The child was managed in isolation with careful monitoring.

The patient’s fever resolved over the next 48–72 h, and her clinical status improved significantly. No bleeding events occurred. By day 5 of hospitalization, her hemoglobin had risen to 7.4 g/dL (post-transfusion) and continued to increase as erythropoiesis recovered. Her platelet count climbed to 89 × 10^3^ μL without additional transfusions. Given the rapid platelet recovery by day 5 without bleeding, hematology consensus favored transient viral suppression over malignancy, recognizing that influenza-induced marrow suppression is typically transient and was already showing improvement. The child remained hemodynamically stable and began feeding better.

By the second week of illness, laboratory values had largely normalized (hemoglobin 9.9 g/dL, platelets 176 × 10^3^ μL at discharge). She was discharged after 8 days in the hospital, alert, afebrile, and well-feeding. Outpatient follow-up was arranged to monitor the patient’s hematologic parameters and growth trajectory, with reassessment for subclinical bone marrow disease or nutritional deficiencies if cytopenias persist or recur, or if new clinical concerns arise. The parents were informed of the advantages of influenza vaccination and were advised to pursue catch-up influenza vaccination after her recovery.

## 3. Discussion

This case illustrates a rare but serious complication of influenza B infection: severe anemia and thrombocytopenia due to apparent transient bone marrow suppression. The hematological abnormalities were likely triggered by proinflammatory cytokines (e.g., interferon-γ, tumor necrosis factor-α), which may have transiently inhibited hematopoiesis. Although specific cytokine levels were not measured in this patient, this mechanism is consistent with prior virus-associated bone marrow suppression reports [14,15]. The patient’s clinical course and laboratory findings align with previously reported influenza-associated hematologic abnormalities in children.

Rice and Resar first described transient pancytopenia during influenza A infection in three pediatric cases [14]. In those cases, bone marrow suppression was presumed to be viral, and blood counts normalized spontaneously as the influenza illness resolved [14]. Similarly, a more recent report by Cohen et al. (2020) [15] documented a previously healthy child who developed anemia and neutropenia during an influenza A infection [15]—a presentation paralleling our patient’s anemia and thrombocytopenia during influenza B. Both cases reflect influenza’s capacity to disrupt bone marrow function even in the absence of predisposing conditions. In our case of influenza B, as in those reports, the cytopenias improved with supportive care and antiviral therapy, suggesting transient influenza-mediated marrow suppression rather than primary bone marrow failure disorder. We did not perform bone marrow aspiration because of the rapid improvement, consistent with the conservative management approach in prior reports when viral marrow suppression is strongly suspected [14,15].

However, the patient’s weight and length were below the fifth percentile, raising concern for potential chronic undernutrition or an underlying condition affecting hematopoiesis or immune function. The caregivers did not report feeding difficulties or socioeconomic factors. Cow’s milk protein allergy was excluded by negative specific IgE testing. Immunologic evaluation, including an immunogram, was normal, reducing the likelihood of a primary immune deficiency. At the time of presentation, no clinical features suggested a syndromic or genetic bone marrow failure disorder. Besides her weight and length being below the fifth percentile, iron deficiency (low ferritin with microcytic anemia) was also found, indicating chronic malnutrition. Malnutrition, even of mild-to-moderate degree, can impair immune function and hematopoiesis [21]. Iron deficiency anemia would lower her baseline hemoglobin reserve, making the drop to 3.1 g/dL more catastrophic. Moreover, iron deficiency and other micronutrients compromise the bone marrow’s capacity to respond to stress and may exacerbate virus-induced cytopenias. A retrospective study found that 6.7% of children hospitalized with influenza had iron deficiency anemia, and its presence was significantly associated with more severe disease courses [22]. We speculate that the child’s suboptimal nutritional state and iron deficiency amplified the hematologic impact of the influenza B infection. During any severe viral illness, a malnourished host is at higher risk of adverse outcomes due to an inadequate compensatory response [23]. This underscores the need to consider nutritional evaluation and support in children who experience an unusually severe course of a common infection. Consistent with this, children with underlying chronic illnesses or nutritional deficits have been observed to suffer more pronounced complications of influenza B [23,24]. Addressing her nutritional needs was part of the overall management plan after the acute phase, and continued follow-up was recommended to monitor growth and reassess if new signs emerged.

Influenza infection can also precipitate hemolytic and thrombotic complications. Bitzan and Zieg reviewed thrombotic microangiopathies (HUS and thrombotic thrombocytopenic purpura, TTP) associated with influenza [17]. While our patient did not meet the full criteria for thrombotic microangiopathy (TMA), influenza-associated HUS in children has been documented [25], reinforcing that influenza can precipitate such thrombotic microangiopathic complications. These syndromes are characterized by microangiopathic hemolytic anemia, thrombocytopenia, and organ injury. Our patient did not meet the clinical or laboratory criteria for HUS/TTP; for example, she had no evidence of hemolysis or renal impairment. However, the presence of severe anemia and thrombocytopenia in an influenza infection could be viewed as part of a spectrum of influenza-induced endothelial and hematopoietic insult. In our case, the negative Coombs test and absence of schistocytes effectively ruled out a microangiopathic process like HUS/TTP as the cause of her cytopenias.

There are fewer published cases of influenza B causing severe bone marrow failure, but emerging reports underscore its potential to result in life-threatening complications. McGraw et al. reported a case of atypical HUS (aHUS) triggered by influenza B in a pediatric patient, which involved simultaneous hemolytic anemia, thrombocytopenia, and acute kidney injury [26]. Another report by van Hoeve et al. (2017) [27] documented atypical HUS associated with influenza B [27]. Although our patient did not develop hemolysis or renal dysfunction, these cases highlight that influenza B can act as a potent systemic stressor, inciting severe hematologic and endothelial complications. Furthermore, an adult case report described influenza B presenting with bicytopenia (leukopenia and thrombocytopenia), which resolved with oseltamivir therapy [28]. These reports and our case demonstrate that influenza B is not always a benign illness in children; it can occasionally provoke life-threatening hematologic manifestations.

Influenza A infection has been more widely linked to hematologic complications, providing pathophysiologic parallels. During the 2009 H1N1 influenza A pandemic, cases of pediatric pancytopenia and bone marrow aplasia were reported [29]. In one single-center study of children with pandemic H1N1 influenza, 25.8% had leukopenia and 19.4% had thrombocytopenia at presentation [30]. Our patient’s influenza B infection elicited a similarly severe hematologic response (albeit without leukopenia). This suggests that the capacity of influenza viruses to cause cytopenias is not strictly dependent on whether they are type A or B but may relate to host factors and the intensity of the infection. Notably, despite the severity of her cytopenias, our patient did not develop a hyperinflammatory syndrome such as hemophagocytic lymphohistiocytosis (HLH); her inflammatory markers had normal values, and there were no clinical signs of HLH.

Influenza can also trigger immune-mediated hematologic disorders [29]. Viruses are known precipitants of idiopathic thrombocytopenic purpura (ITP) in children; approximately two-thirds of pediatric ITP cases follow a viral illness [31]. Influenza (including type B) has been implicated as one such trigger in case reports [32]. For example, Jung et al. (2015) described a 5-year-old boy who developed acute ITP (platelet count 13 × 10^3^ μL) during an influenza B infection, representing the first documented influenza-associated ITP in the English literature [32]. That child recovered with intravenous immunoglobulin and corticosteroids, suggesting an infection-triggered autoimmune mechanism. In our patient, however, the concurrent anemia (and involvement of multiple cell lines) made primary post-influenza ITP unlikely. In classic post-viral ITP, usually only platelets are severely affected (with the rest of the blood counts normal), and anemia would appear only secondary to hemorrhage, which was not the case here. Indeed, our patient’s platelet count improved rapidly with recovery from influenza and supportive therapy, and she had no clinical evidence of bleeding. Nonetheless, we administered corticosteroids empirically as a precaution, given her extremely low platelet count and the potential (albeit uncertain) contribution of immune-mediated platelet destruction.

Similarly, in rare instances, influenza infection can precipitate autoimmune hemolytic anemia (AIHA). Bandyopadhyay et al. reported a 16-month-old girl who developed warm AIHA (Coombs-positive for IgG) following an influenza A(H1N1) infection, requiring corticosteroids and IV immunoglobulin for recovery [33]. In our case, there were no signs of autoimmune hemolysis; the direct antiglobulin test was negative, and haptoglobin, bilirubin, LDH, and uric acid were normal, without hemoglobinuria and a low reticulocyte count. This indicates that the anemia was due to deficient production rather than peripheral destruction. Another rare influenza-related hemolytic complication is paroxysmal cold hemoglobinuria (PCH). Maslak et al. described life-threatening PCH in an 8-year-old boy precipitated by an influenza A infection [34]. PCH is a form of autoimmune hemolysis caused by Donath–Landsteiner antibodies. Although PCH and AIHA are uncommon, their occurrence with influenza underscores how this virus can trigger immune dysregulation, leading to the destruction of red cells and platelets. These reports highlight the need for clinicians to remain vigilant for hematologic sequelae in children with influenza who develop unexplained anemia, thrombocytopenia, or other cytopenias beyond the usual course of a viral illness.

Bote et al. (2021) [35] demonstrated that human platelets can internalize influenza virus and become activated, indicating a potential immunological mechanism where platelets contribute to innate immune sensing and may amplify inflammatory responses during influenza infection [35]. This case illustrates a rare but serious hematologic complication of influenza B—severe anemia and thrombocytopenia—likely due to transient bone marrow suppression.

Interestingly, similar hematologic abnormalities have been documented in pediatric COVID-19 cases, particularly AIHA, as described by Zama et al. [36]. In their report, children developed profound anemia (hemoglobin < 4 g/dL) with immune-mediated red cell destruction and, in some cases, concurrent thrombocytopenia, consistent with Evans syndrome. Unlike our patient, in whom anemia appeared to have resulted from marrow suppression without hemolysis (negative Coombs test, low reticulocyte count), the COVID-19-associated cases involved autoimmune hemolysis, as evidenced by a positive direct antiglobulin test (DAT) and elevated bilirubin and LDH.

Both influenza and SARS-CoV-2 can trigger severe hematologic responses through distinct yet sometimes overlapping mechanisms: direct bone marrow suppression, cytokine-induced myelosuppression, or immune-mediated cytopenias. Our case and the COVID-19 AIHA cases illustrate how respiratory viruses, though primarily affecting the lungs, can exert systemic effects, including significant hematologic injury. These parallels emphasize the need for vigilance in recognizing and managing the hematologic complications of viral infections, regardless of the specific pathogen involved.

In summary, influenza (A or B) can cause cytopenias through multiple mechanisms: direct bone marrow suppression, cytokine-mediated myelosuppression, and immune-mediated destruction of blood cells [37,38]. Early identification of cytopenias in the context of influenza is critical, as it prompts timely supportive interventions and appropriate evaluation for alternative causes. In severe cases, aggressive supportive therapy may be necessary to prevent severe outcomes [39], including transfusions and immunomodulatory treatment, which may be warranted to improve outcomes [40]. In our patient, prompt blood product transfusions, antiviral therapy, and corticosteroids led to rapid hematologic recovery.

Although Hanula et al. [41] found that oseltamivir was not significantly associated with reduced hospitalization rates in a general outpatient population, it is important to note that the analysis did not specifically evaluate treatment initiated beyond the 48 h window, nor did it focus on patients with severe or progressive illness. In contrast, the U.S. Centers for Disease Control and Prevention (CDC) explicitly recommends starting antiviral treatment even after 48 h in certain populations. Particularly vulnerable patients are those who are hospitalized, have severe or complicated illness, or are at higher risk for influenza-related complications (e.g., young children, the elderly, immunocompromised individuals) [9]. Observational studies and clinical experience support that delayed antiviral therapy may still confer meaningful benefit in these high-risk groups. Therefore, while Hanula’s meta-analysis informs general outpatient practice, it does not contradict CDC-endorsed treatment in select patients beyond the typical 48 h window.

Severe cytopenias during influenza have been associated with worse outcomes in hospitalized patients [16], so recognizing this complication early may improve management. In our patient, the decision to forego an immediate bone marrow biopsy proved appropriate because she showed clear improvement with conservative management. In a hemodynamically stable child who is improving, it is reasonable to defer invasive procedures that would otherwise be indicated for pancytopenia. Our patient’s bone marrow function began to recover within days of antiviral and supportive therapy, which strongly supported the presumptive diagnosis of transient virus-induced marrow suppression. This approach is supported by prior observations that influenza-associated cytopenias often resolve spontaneously as the infection clears [14]. Of course, close monitoring is essential; had she not demonstrated hematologic recovery as expected, a bone marrow examination would have been imperative to exclude diagnoses like leukemia or aplastic anemia. Avoiding an unnecessary invasive procedure spared the child additional risk and discomfort.

Influenza infection rarely precipitates transient pancytopenia or bicytopenia in children, leading to divergent management strategies in reported cases [42]. Some clinicians advocate for early bone marrow examination to exclude critical diagnoses (leukemia, aplastic anemia, HLH), especially since multi-lineage cytopenia often warrants thorough evaluation [43]. For instance, Jung et al. describe a child with severe influenza B-associated thrombocytopenia who underwent a bone marrow aspirate/biopsy to confirm post-viral immune thrombocytopenic purpura, with the marrow showing normal cellularity (no malignancy or hemophagocytosis) [32]. Similarly, influenza-triggered HLH has been reported, where marrow aspirate revealed hemophagocytes, securing the diagnosis and guiding urgent therapy [44]. However, other case series emphasize that influenza-associated cytopenias often resolve spontaneously as the infection clears [42]. In such cases, invasive workup was deferred in favor of close monitoring, given reassuring features—a normal peripheral smear (absence of blasts), no organomegaly, and an appropriate reticulocyte response with improving counts—all suggesting transient viral marrow suppression. Indeed, many viral cytopenias (including influenza) show a benign, self-limited course with recovery of blood counts in about 1–2 weeks [42]. Our patient’s course mirrors these reports: despite severe anemia and thrombocytopenia from influenza B, she improved rapidly on antiviral and supportive care without bone marrow biopsy. This conservative approach spared her an invasive procedure and its risks, but we acknowledge the trade-off—the mechanism remained presumptive without histopathologic confirmation. In summary, early bone marrow evaluation offers diagnostic certainty. It is indispensable if an occult malignancy or HLH is suspected. In contrast, watchful waiting can be justified in a stable, recovering child, aligning with recent viral cytopenia case reports (including influenza and SARS-CoV-2-associated cytopenias [36]) that underscore individualized decision-making based on clinical and laboratory trajectory.

**Figure 2:**
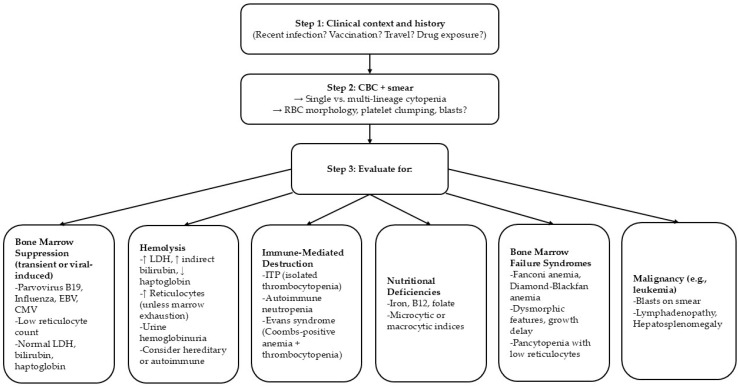
Differential diagnosis of viral-induced thrombocytopenia.

Our patient’s inflammatory markers (CRP, procalcitonin) were normal despite her severe illness. This relatively blunted systemic inflammatory response provided a clue to the etiology of the cytopenias. In bacterial sepsis or hyperinflammatory syndromes like HLH, one would expect markedly elevated inflammatory markers accompanying cytopenias. In this case, the absence of such findings supports a primary viral effect on the bone marrow rather than a secondary cytokine storm or overwhelming sepsis. Likewise, the normal haptoglobin and LDH levels and negative Coombs test argue against ongoing hemolysis as a major contributor to the anemia. Thus, the laboratory profile helped distinguish isolated viral marrow suppression from other diagnoses such as HLH (which often features high ferritin and cytopenias) or thrombotic microangiopathies like aHUS/TTP (which typically show hemolysis with fragmented cells on smear). The following steps must be considered in the differential diagnosis of thrombocytopenia during viral infections (Figure 2).

From a public health perspective, this case highlights the critical role of influenza vaccination in children. Neither our patient nor her family members had received the influenza vaccine for the current season. Annual influenza vaccination is recommended for all children ≥ 6 months of age and has been proven to significantly reduce the risk of severe illness and complications [45]. While profound marrow suppression is an exceedingly rare complication of influenza, it is potentially life-threatening and could presumably be prevented by preventing the influenza infection in the first place. Our patient’s young age (under 2 years) and her nutritional vulnerability placed her at higher risk for severe disease, precisely the population in which influenza vaccination is most strongly indicated [23]. Educating families that influenza can do more than cause “just a cold”, which can lead to severe organ-specific complications (including hematologic failure), may improve vaccine uptake. Achieving high vaccination coverage within households and the community (herd immunity) is vital to protecting infants and toddlers who may not mount a robust immune response even if vaccinated. It is conceivable that, if our patient’s family had been immunized, her exposure to influenza might have been minimized, or the severity mitigated.

## 4. Conclusions

In pediatric patients with unexplained cytopenias, particularly during seasonal outbreaks, differential diagnosis should consider influenza B infection, given its potential to mimic hematologic malignancies. Rapid virologic testing is essential to avoid delayed diagnosis and unnecessary interventions. Second, adherence to evidence-based thresholds for transfusion (Hb < 7 g/dL or platelets < 50 × 10^3^/µL) should prompt immediate hematologic evaluation, irrespective of leukopenia, to mitigate complications. Our case report highlights the crucial role of annual influenza vaccination in pediatric populations, which is proven to reduce severe morbidity, ICU admissions, and the risk of life-threatening hematologic consequences. These findings underscore the two key components of diagnostic vigilance and vaccination in optimizing results.

## Figures and Tables

**Table 1:** Daily evolution and interventions.

Day	Clinical Findings	Interventions
1–3	Fever (39.4 °C), dry cough, vomiting	Supportive care at home
4	Pallor, lethargy	RBC/platelet transfusions, oseltamivir
5	Afebrile	Corticosteroids, monitoring
8	No notable clinical findings	Discharged with outpatient follow-up

[Normal values: Plt (platelets) 150–450 × 10^3^/µL; Hb (hemoglobin) 10.5–14 g/dL. RBC—red blood cell].

**Table 2:** Daily evolution of the lab results.

Bloodwork	Day 1	Day 2	Day 3	Day 4	Day 5	Day 6	Day 7
WBC (10^3^ μL)	5.66	8.2	9.49	11.27	10.93	8.5	7.72
ANC (10^3^ μL)	1.9	2.12	2.18	2.17	2.03	2.36	2.68
ALC (10^3^ μL)	3.47	5.4	6.53	7.07	6.89	5.6	4.66
AMC (10^3^ μL)	0.12	0.54	0.57	0.60	0.47	0.40	0.35
RBC (10^6^ μL)	2.16	2.80	3.85	3.60	4.36	4.95	4.81
Hb (g/dL)	3.1	7.2	8	7.4	8.9	10.2	9.9
HCT %	12.4	20.5	27.2	27.5	26.7	35.3	34.6
MCV (fL)	55	69	70	72.5	69.2	71.2	71.9
MCH (pg)	14.6	19.5	21.8	20.1	20	20.6	20.6
MCHC (g/dL)	25.3	28.2	28.4	28.5	29	30.2	28.6
PLT (10^3^ mL)	15	48	78	89	110	153	176
ARC (10^6^ μL)	0.0146					0.2260	
RET %	0.68	1.5	2.8	3.7	4.5	4.56	5.4

[WBCs—white blood cells; ANC—absolute neutrophil count; ALC—absolute lymphocyte count; AMC—absolute monocyte count; RBCs—red blood cells; Hb—hemoglobin; HCT—hematocrit; MCV—mean corpuscular volume; MCH—mean corpuscular hemoglobin; MCHC—mean corpuscular hemoglobin concentration; PLT—platelet count; ARC—absolute reticulocyte count; RET %—reticulocyte percentage].

**Table 3:** Hemolysis markers.

Hemolysis Marker	Tested	Result/Status
LDH	Yes	Normal (329 U/L)
Total bilirubin	Yes	Normal (0.18)
Direct bilirubin	Yes	0.18
Indirect bilirubin	Yes	0.01
Haptoglobin	Yes	Normal (60 mg/dL)
RET %	Yes	0.68% (inappropriately low for anemia)
ARC	Yes	0.0146 × 10^6^/μL (low)
Uric acid	Yes	Normal (2.2 mg/dL)
Urine hemoglobin	Yes	Negative (no hemoglobinuria)

## Data Availability

All data supporting this case report are included in the manuscript.

## Abbreviations

The following abbreviations are used in this manuscript:

aHUS	Atypical hemolytic uremic syndrome
AIHA	Autoimmune hemolytic anemia
ALC	Absolute lymphocyte count
AMC	Absolute monocyte count
ANC	Absolute neutrophil count
ARC	Absolute reticulocyte count
COVID-19	Coronavirus disease 2019
DAT	Direct antiglobulin test
Hb	Hemoglobin
HCT	Hematocrit
HLH	Hemophagocytic lymphohistiocytosis
HUS	Hemolytic uremic syndrome
IDA	Iron deficiency anemia
ITP	Immune thrombocytopenic purpura
IV	Intravenous
MCH	Mean corpuscular hemoglobin
MCHC	Mean corpuscular hemoglobin concentration
MCV	Mean corpuscular volume
PCH	Paroxysmal cold hemoglobinuria
Plt	Platelet
PLT	Platelet count
RBC	Red blood cell
RET %	Reticulocyte percentage
SARS-CoV-2	Severe acute respiratory syndrome coronavirus 2
TMA	Thrombotic microangiopathy
TTP	Thrombotic thrombocytopenic purpura
WBC	White blood cell
